# Supplementary material for: Near-strain-free anode architecture enabled by interfacial diffusion creep for initial-anode-free quasi-solid-state batteries
Source: Nat Commun. 2024 Apr 27;15:3586. doi: 10.1038/s41467-024-48021-w (PMC11055892; doi:10.1038/s41467-024-48021-w)
Supplement: Supplementary file 1 — Supplementary Information [file 41467_2024_48021_MOESM1_ESM.pdf]

Supplementary information

Near-strain-free anode architecture enabled by interfacial diffusion creep for initial-anode-free quasi-solid-state batteries

Kwang Hee Kim[1†], Myung-Jin Lee[2†], Minje Ryu[1†], Tae-Kyung Liu[1], Jung Hwan Lee[1], Changhoon Jung[3], Ju-Sik Kim[2*] and Jong Hyeok Park[1*]

[1] Department of Chemical and Biomolecular Engineering, Yonsei University, 50 Yonsei-ro, Seodaemun-gu, Seoul 03722, Republic of Korea.

[2] Battery Material TU, Samsung Advanced Institute of Technology, 130, Samsung-ro, Yeongtong-gu, Suwon-si, Gyeonggi-do, 16678, Republic of Korea.

[3] Analytical Engineering Group, Samsung Advanced Institute of Technology, 130, Samsung-ro, Yeongtong-gu, Suwon-si, Gyeonggi-do, 16678, Republic of Korea.

* Correspondence and requests for materials should be addressed to: jusik.kim@samsung.com, and lutts@yonsei.ac.kr.

†These authors are contributed equally.

Supplementary Table 1. Summary of thickness change of various pouch-type full-cells. ($\Delta T_{initial}$ = Average thickness change compared to initial thickness, ΔT_{cycle} = Average thickness change during charging/discharging cycle)

Sample	$\Delta T_{initial}$ (μm)	ΔT_{cycle} (μm)
Ag-C/Li	42.1	16.3
Pre_Li_Ag-C/Li	15.1	11.6
**Pre_Li_Ag-C/TiN NT**	**3.5**	**4.3**

Supplementary Table 2. Fitting parameters determined from CNLS fitting of the measured impedance spectra in Supplementary Fig. 18.

[Symmetric cell (Pristine)]

Fitting parameters	Anode			
	Cu		TiN NT	
	Fit values	Error (%)	Fit values	Error (%)
R_b [Ω cm^2]	18.67	0.36	18.85	0.42
R_1 [Ω cm^2]	5.76	2.52	3.96	2.81
CPE_1-T	6.12×10^{-6}	19.17	3.17×10^{-5}	20.44
CPE_1-n	0.86	2.38	0.77	3.00
chi-square	5.1×10^{-4}		9.68×10^{-4}	

[Symmetric cell (After cycle)]

Fitting parameters	Anode			
	Cu		TiN NT	
	Fit values	Error (%)	Fit values	Error (%)
R_b [Ω cm^2]	18.08	0.57	18.44	0.48
R_1 [Ω cm^2]	9.03	1.65	5.56	2.27
CPE_1-T	1.36×10^{-5}	14.26	2.92×10^{-5}	17.94
CPE_1-n	0.75	2.02	0.74	2.70
chi-square	1.78×10^{-3}		1.45×10^{-3}	

Supplementary Table 3. Fitting parameters determined from CNLS fitting of the measured impedance spectra in Fig. 5e and Supplementary Fig.20.

[60 °C]

Fitting parameters	Anode			
	Cu		TiN NT	
	Fit values	Error (%)	Fit values	Error (%)
R_b [Ω cm^2]	7.34	0.11	5.0	0.15
R_1 [Ω cm^2]	14.10	0.93	12.75	0.93
R_2 [Ω cm^2]	2.54	2.76	2.41	2.35
$R_{ct} = R_1 + R_2$ [Ω cm^2]	16.64		15.16	
CPE_1-T	1.85×10^{-5}	2.54	1.10×10^{-5}	2.67
CPE_1-n	0.76	0.33	0.87	0.31
W_R [Ω cm^2]	14.44	10	63.87	11.22
W-T	0.076	10.19	0.25	13.82
W-n	0.40	0.79	0.37	1.38
CPE_2-T	4.8×10^{-6}	15.85	2.44×10^{-6}	16.07
CPE_2-n	0.89	4.05	0.95	3.44
chi-square	1.35×10^{-4}		2.19×10^{-4}	

[25 °C]

Fitting parameters	Anode			
	Cu		TiN NT	
	Fit values	Error (%)	Fit values	Error (%)
R_b [Ω cm^2]	31.06	0.16	22.01	0.2
R_1 [Ω cm^2]	25.98	4.39	12.34	3.98
R_2 [Ω cm^2]	81.39	1.21	49.74	2.79
$R_{ct} = R_1 + R_2$ [Ω cm^2]	107.37		62.08	
CPE_1-T	9.3×10^{-6}	8.95	3.17×10^{-6}	12.6
CPE_1-n	0.73	1.11	0.87	1.48
W_R [Ω cm^2]	61.97	3.85	63.87	3.95
W-T	0.29	5.56	0.25	6.3
W-n	0.40	0.99	0.37	1.92
CPE_2-T	5.75×10^{-6}	2.37	2.44×10^{-6}	5.95
CPE_2-n	0.86	0.07	0.95	1.18
chi-square	3.38×10^{-4}		2.60×10^{-4}	

Supplementary Table 4. Comparison of the electrochemical performances between TiN NT incorporated AFSSB and previously reported garnet-type SE-based SSBs.

Cell configuration	Current density [mA cm^{-2}]	No. of cycles	Operating temperature [°C]	Cathode mass loading [mg cm^{-2}]	Cathode areal capacity [mAh cm^{-2}]	Ref.
TiN NT|Pre_Li_Ag-C/LLZTO|IL/NCM333	1.0	600	25		3.20	This work
Li-In|LLZ:Ta|LCO	0.05	100	50		1.50	S1
Li|Li_3PO_4-LLZTO|NCM523	0.15	120	25	4.30		S2
Li|Ge-modified-garnet|LFP	0.05	100	25		0.10	S3
Li|LLZO|PCE|NCM111	0.05	26	25		0.33	S4
Li|PEGMEA-p-LATP|NCM811	0.1	50	25	1.89		S5
Li|LLZTO-RAT|LCO	0.03	100	30	2.0		S6
Li|ITO-LLZTO|LFP	0.2	100	30	2.0		S7
Li|LLZTO-MCL|LCO	0.05	300	25	2.0		S8
Li|Graphite coated LALZWO|IL/NCM523	0.08	500	25	2.0		S9
LNO10|SN-LLZTO|NCM811	0.36	115	25	2.0		S10
Li|LLZONb|LCO	0.005	100	25	-	-	S11
Li|s@LLAZO(6h)-50PEGDA|NCM333	0.1	250	25	1.5		S12
Li|EM|NCM622	0.5	200	25	2.3		S13
Li|LN-LLZT|LFP	0.1	200	40	2.0		S14
Li|LLZO|LCO	0.006	100	25	1.0		S15
Li|Ga-LLZO|LFP	0.3	50	27	5.0		S16
SUS/PT|LiPON|LCO/Au	0.005	100	25	-	-	S17
Cu|LLZO|PEO-NCA	0.3	50	60		3.0	S18
Cu|LLZTO/PEO-CPE|NCM333	0.2	100	55	-	-	S19
Li|Ag-C/Ag/LLZTO/IL|NCM333	1.6	800	25	-	2.70	S20*

*Reported by Samsung Advanced Institute of Technology (SAIT)

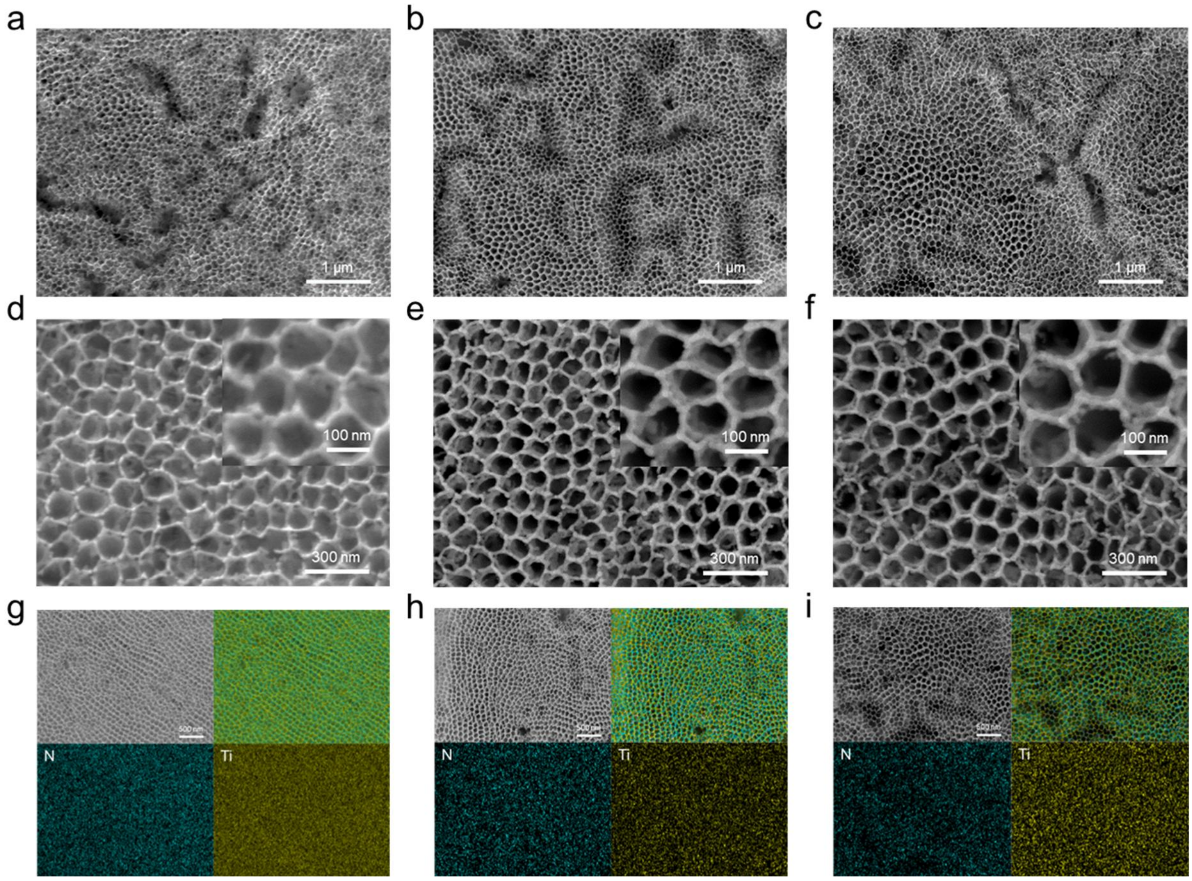

Supplementary Fig. 1. a-c, Low-magnification top-view SEM images of the TiN NT using a) 250 μm, b) 127 μm, and c) 50 μm. **d-f,** Top-view SEM and high-magnification top-view SEM (inset) images of the TiN NT using d) 250 μm, e) 127 μm, and f) 50 μm. **g-i,** EDS images of the TiN NT using g) 250 μm, h) 127 μm, and i) 50 μm.

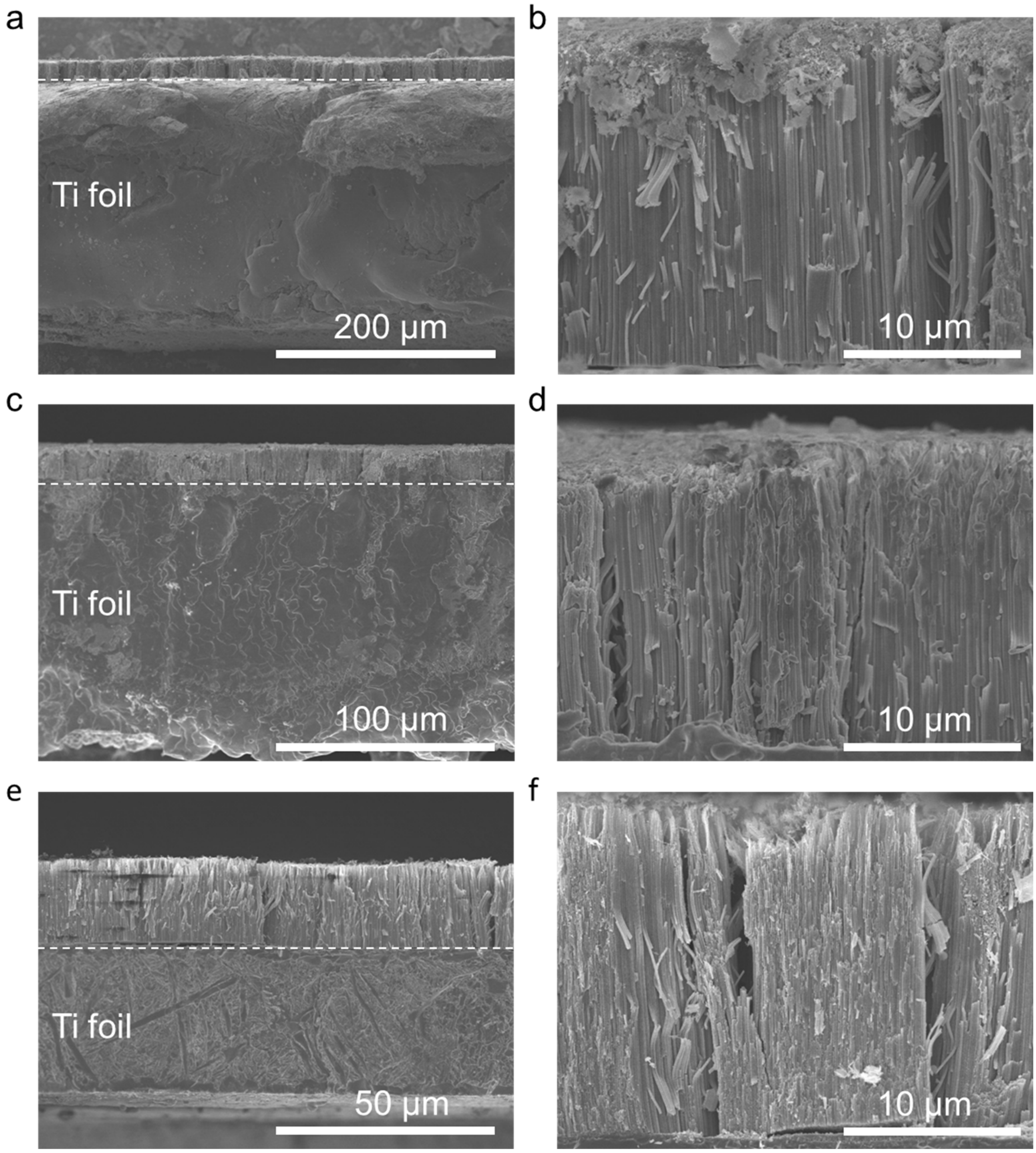

Supplementary Fig. 2. a-f, Cross sectional SEM images of the TiN NT using a), b) 250 μm, c), d) 127 μm, and e), f) 50 μm.

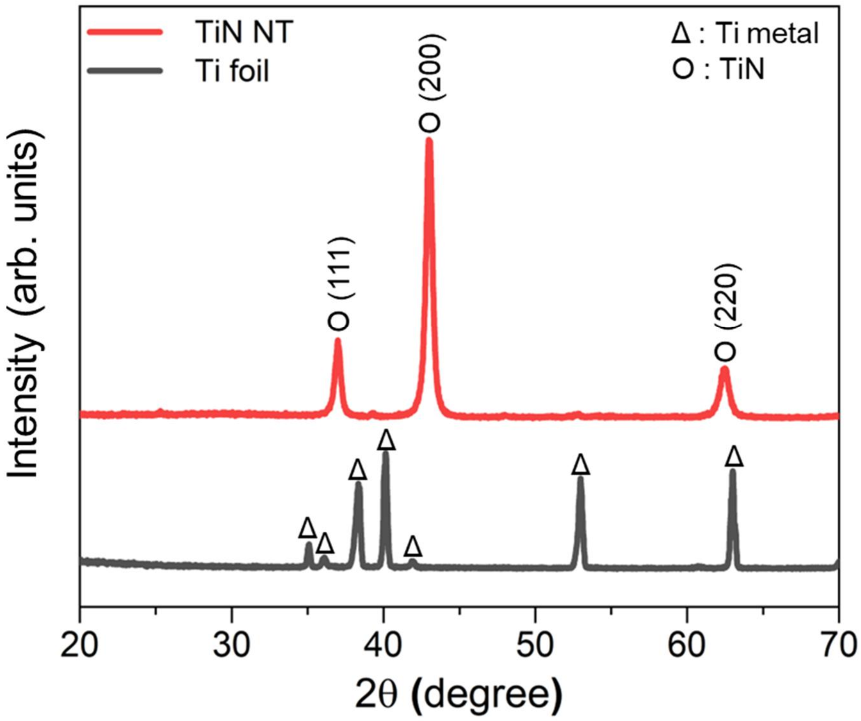

Supplementary Fig. 3. XRD patterns of Ti foil and TiN NT.

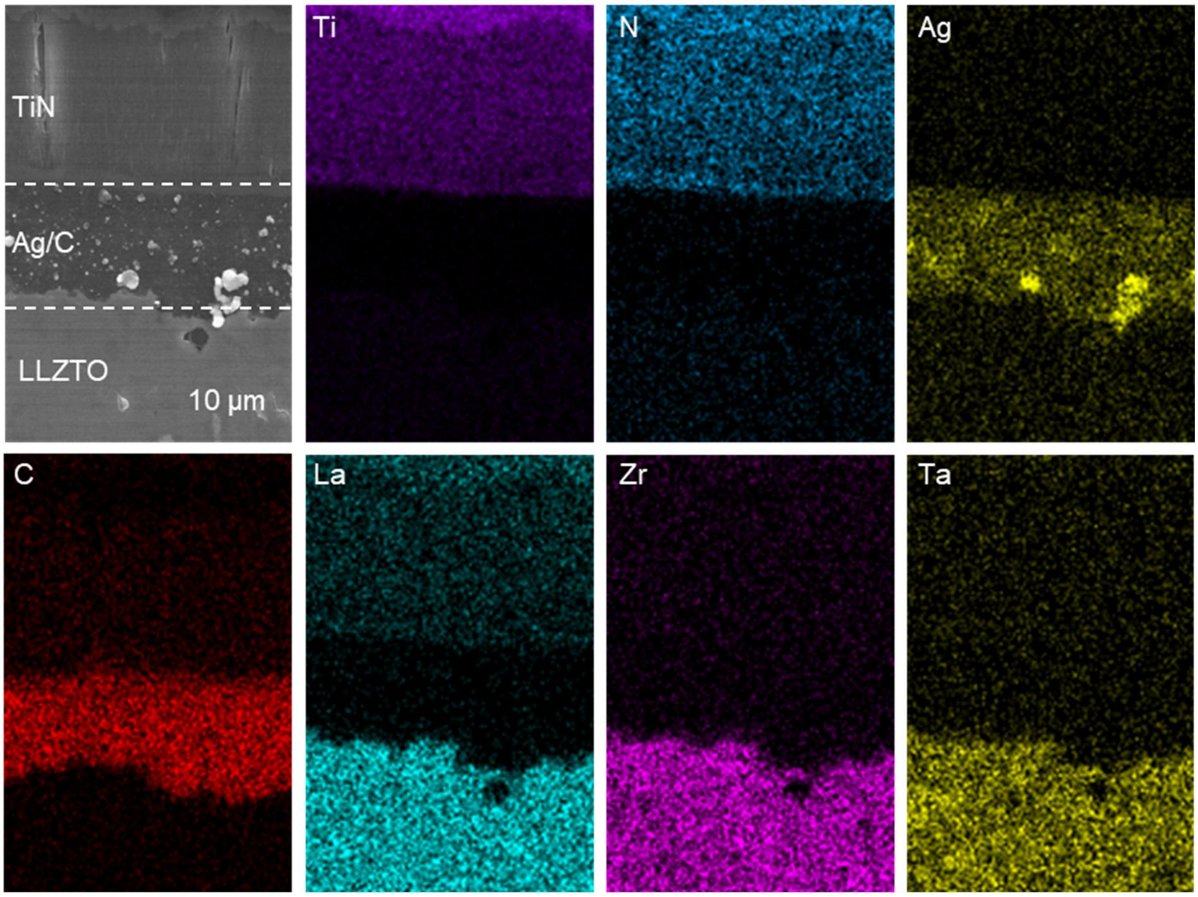

Supplementary Fig. 4. Cross-sectional SEM image and corresponding EDS images of the TiN NT incorporated garnet-type SE based anode-free SSB.

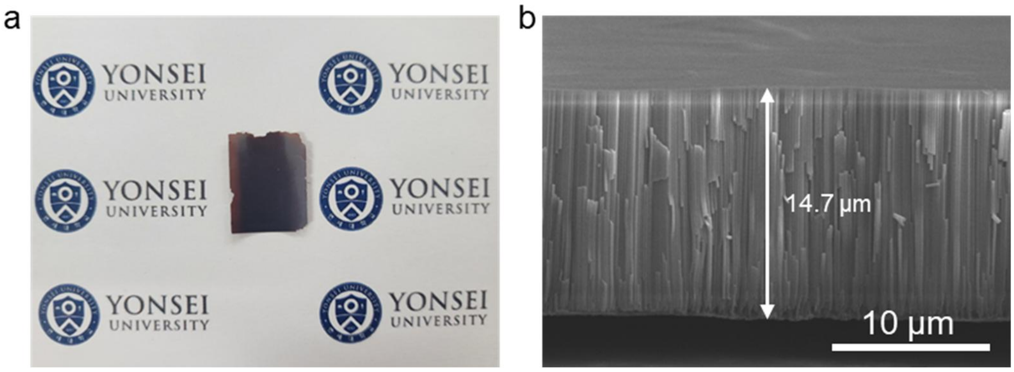

Supplementary Fig. 5. a, Optical image of free-standing TiN NT film. **b,** Cross-sectional SEM image of free-standing TiN NT film.

To calculate the porosity of the TiN NT, a free-standing TiN NT film was first obtained through detachment from the Ti foil. Subsequently, the true density of the TiN NT film was determined through volume and mass measurements. Based on the true density and the theoretical density of TiN, the porosity of TiN NT was calculated to be 78.4%. The porosity calculation is performed using the following equations:

$$\rho\ (true\ density) = \frac{m_{TiN}}{V_{TiN}}$$

$$Porosity\ (\%) = \left(1 - \frac{\rho\ (true\ density)}{\rho_{TiN}\ (theoretical\ density)}\right) \times 100\%$$

Where, V_{TiN} = volume of the free-standing TiN NT film, m_{TiN} = mass of the free-standing TiN NT film, and ρ_{TiN} = theoretical density of the titanium nitride.

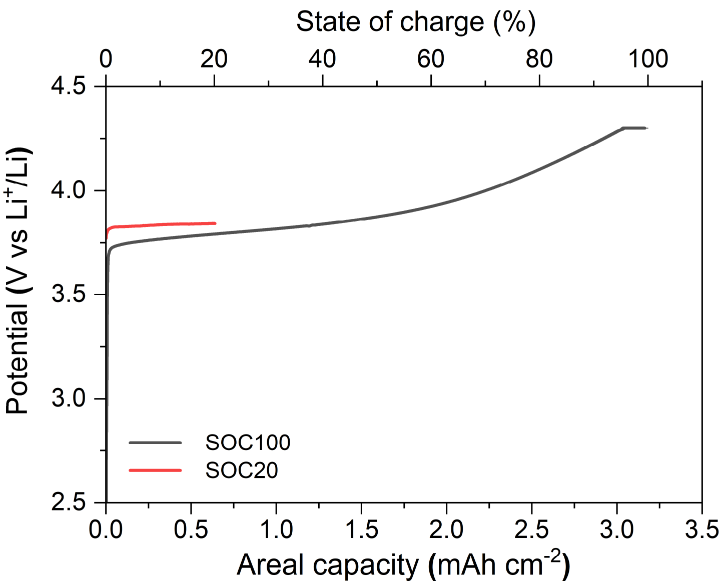

Supplementary Fig. 6. Voltage profiles of the TiN NT incorporated anode-free SSB full-cell at different charged states i.e., SOC 20% and SOC 100%.

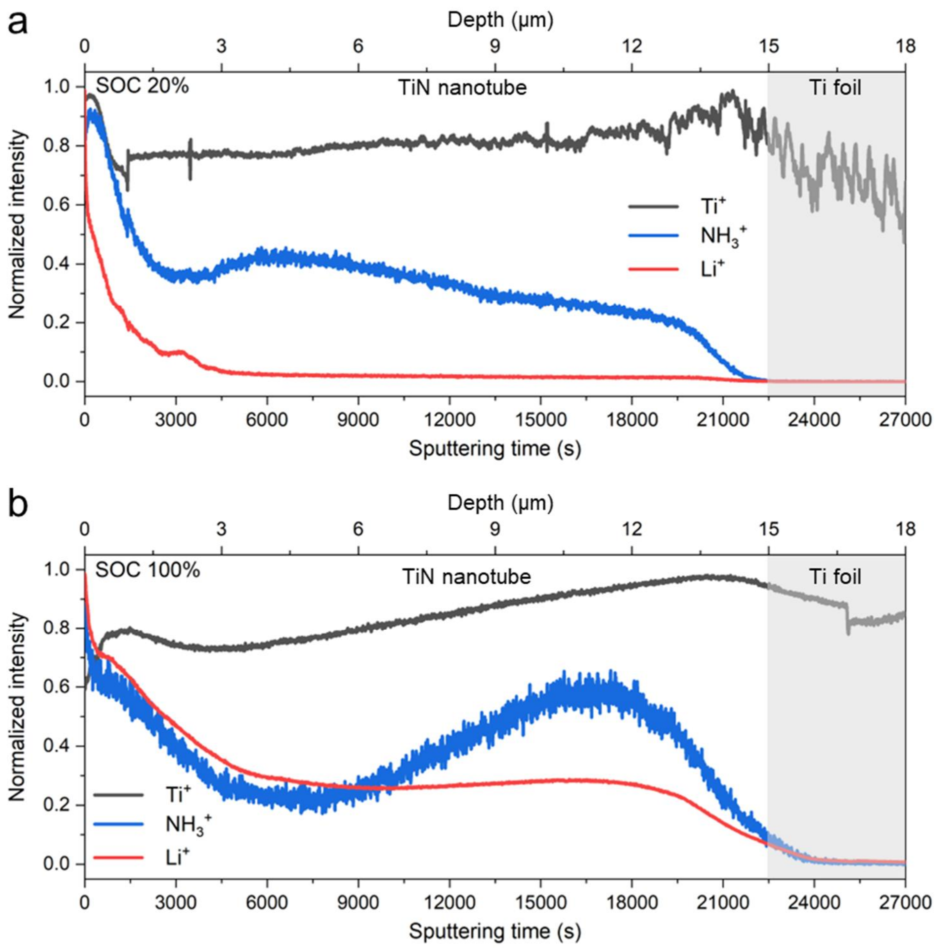

Supplementary Fig. 7. Normalized TOF-SIMS depth profile of various secondary ions for TiN NTs at **a,** 20% SOC and **b,** 100% SOC.

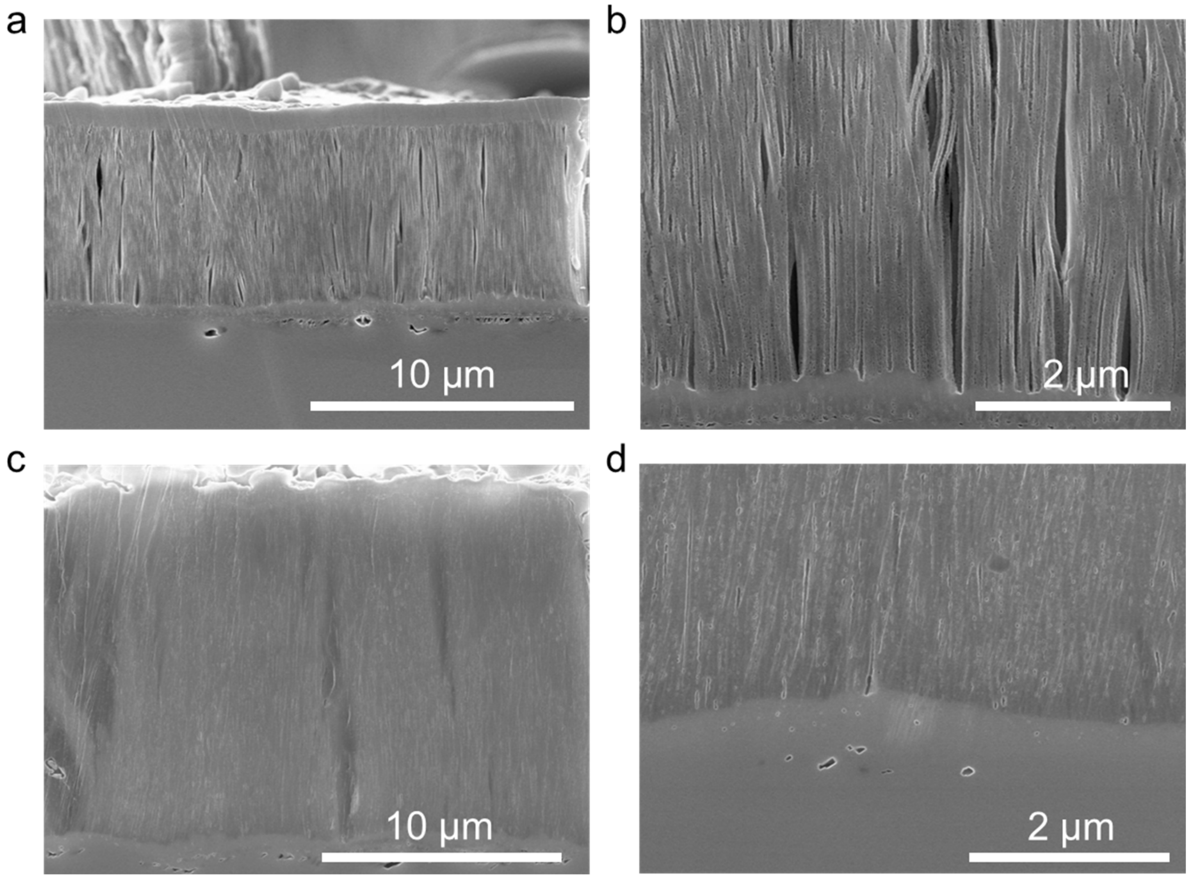

Supplementary Fig. 8. a-d, Cross-sectional SEM images of TiN NT at a), b) SOC 0%, and c), d) SOC 100%.

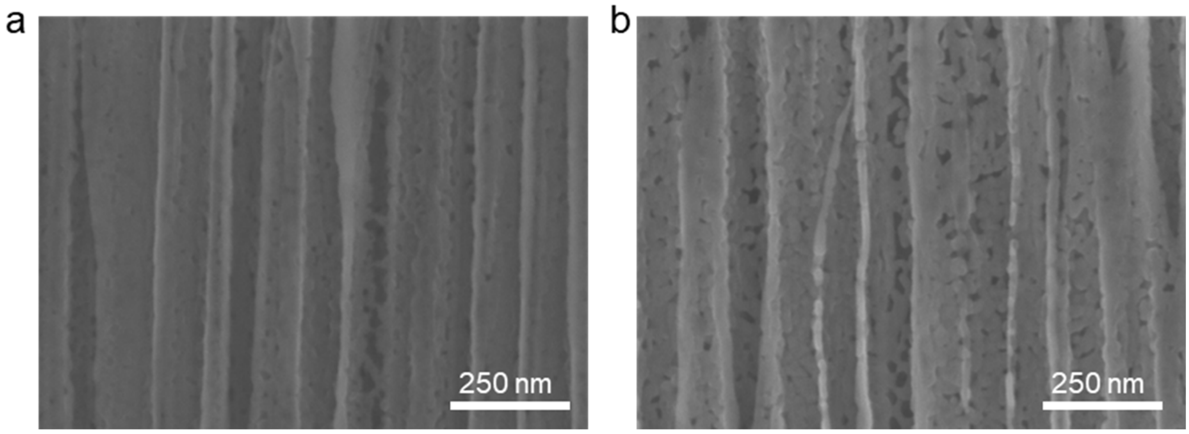

Supplementary Fig. 9. Cross-sectional SEM images of TiN NT at a) before cycling, and b) after cycling.

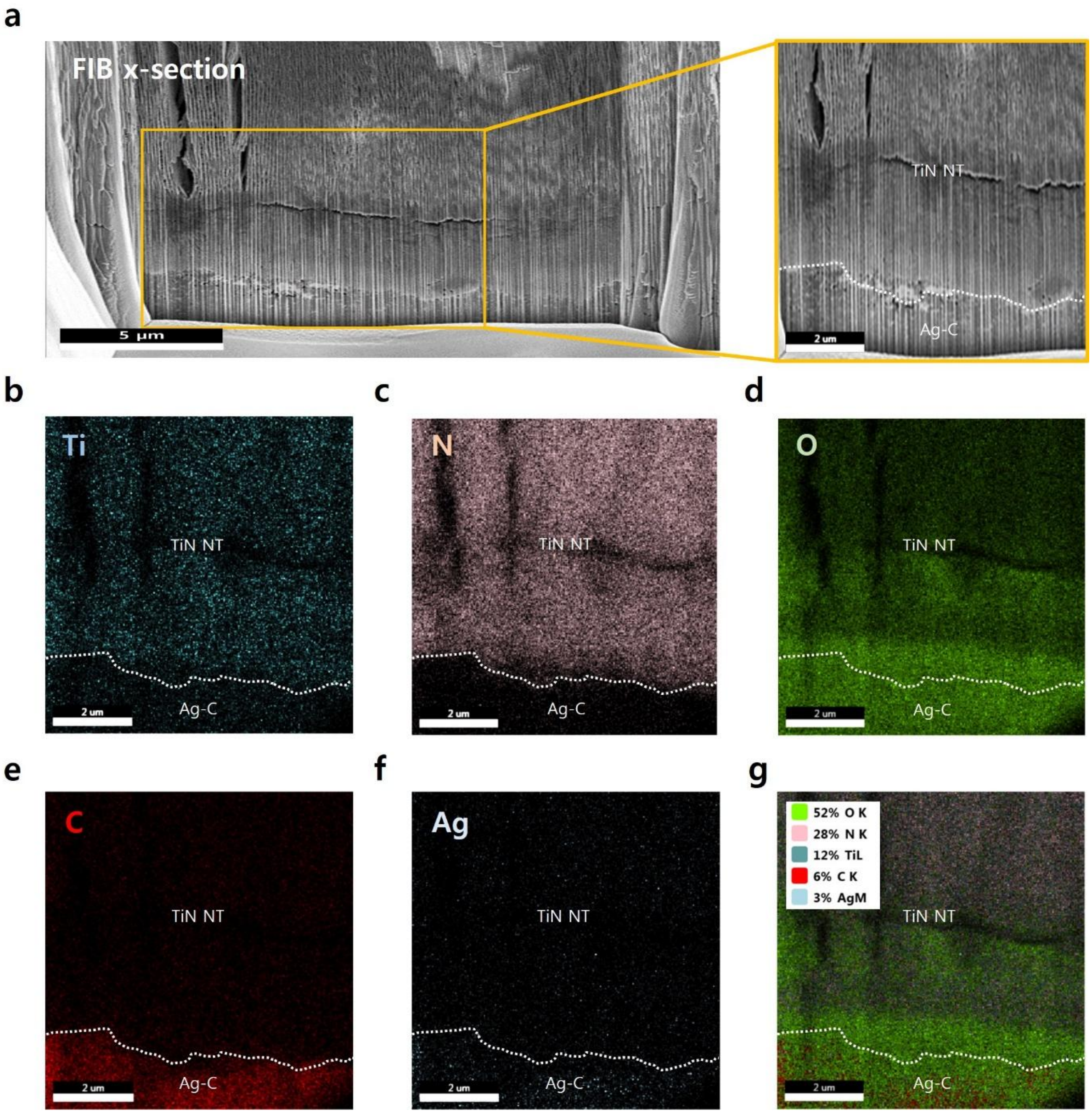

Supplementary Fig. 10. a, Cross-sectional FIB-SEM image, and **b-g,** Corresponding EDS elemental mapping images of the TiN NT-incorporated AFSSB after charging.

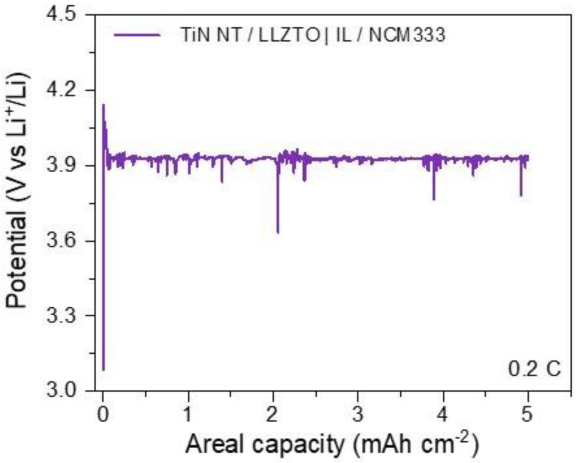

Supplementary Fig. 11. Charge/discharge voltage profile of the TiN NT incorporated full-cell without Ag-C interlayer.

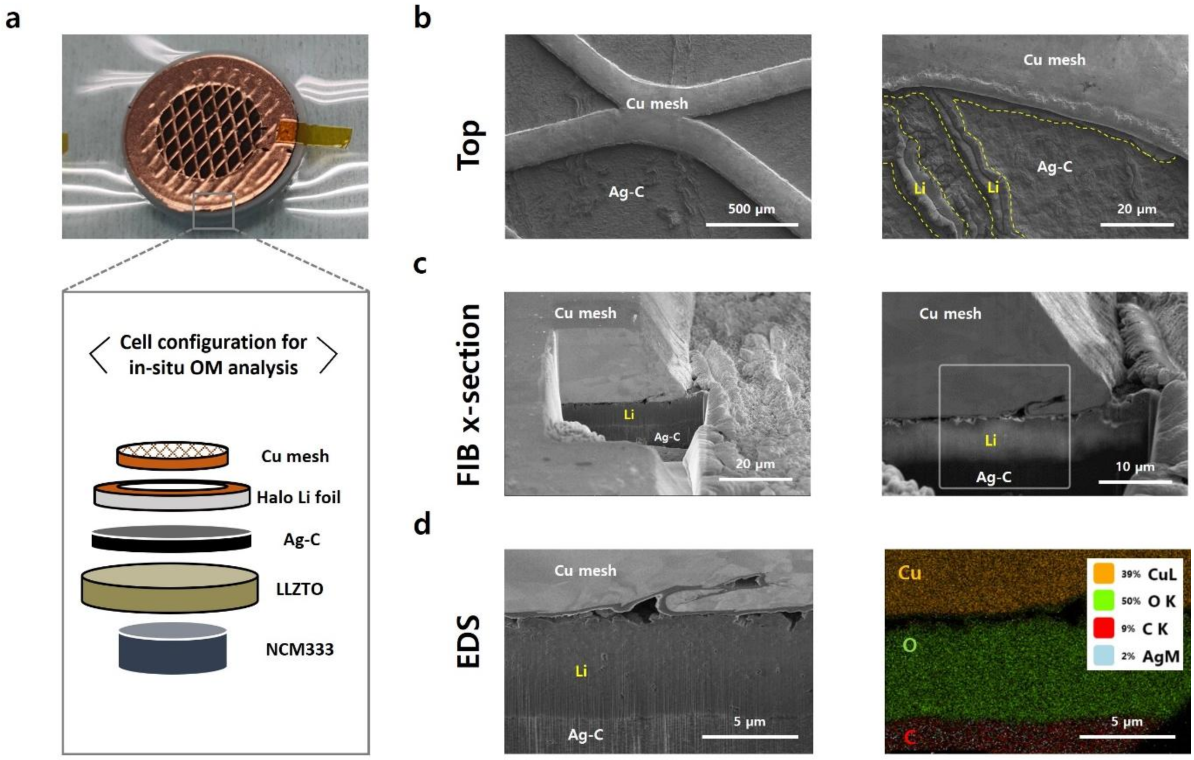

Supplementary Fig. 12. a, Digital image, and schematic of the cell configuration used for in-situ OM analysis. **b,** Top SEM images of the charged cell after the in-situ OM experiment. **c,** Cross-section FIB-SEM images of the edge side of Cu mesh revealing layers of Li metal and Ag-C. **d,** Corresponding EDS elemental mapping image of the marked region.

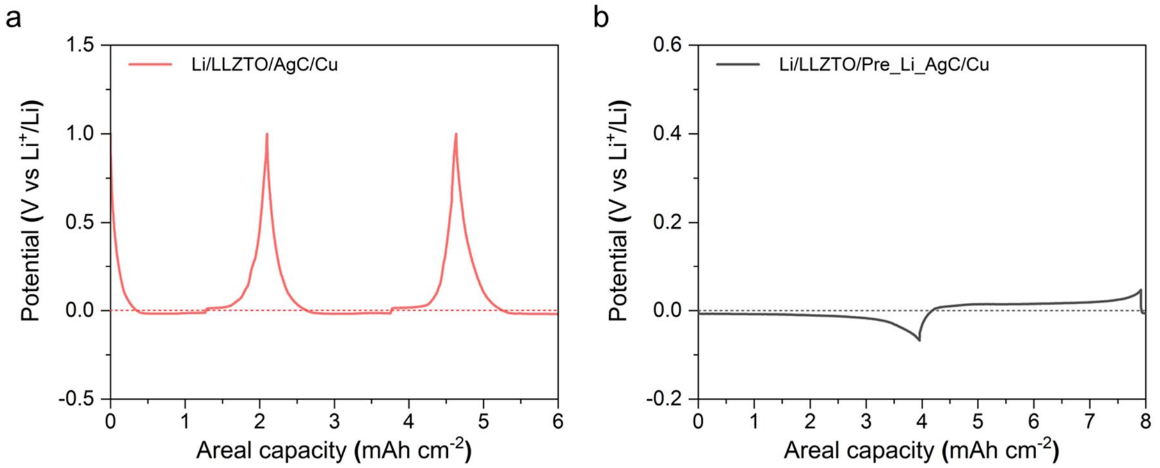

Supplementary Fig. 13. a-b, Voltage profiles of LLZTO solid electrolyte-based Li-metal cells with different interlayers, a) Ag-C and b) pre-lithated Ag-C.

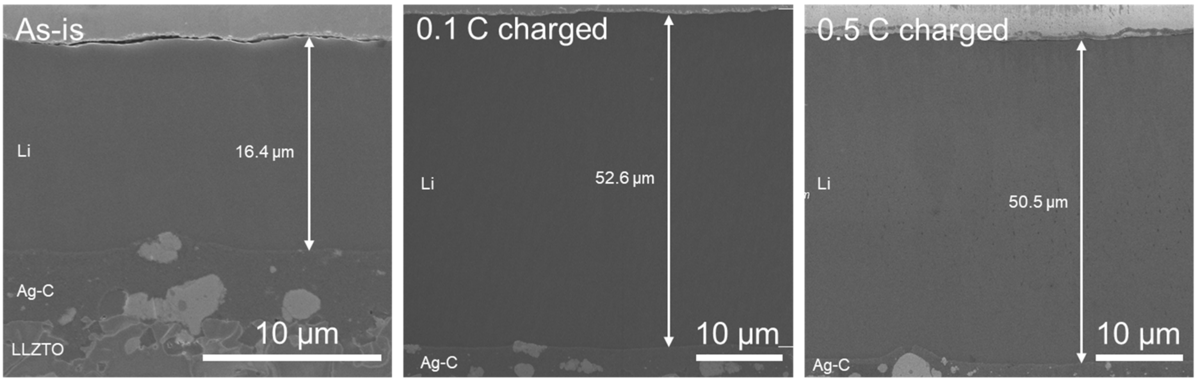

Supplementary Fig. 14. Cross-sectional SEM images of the LLZTO solid electrolyte-based Li-metal cell before and after charging at different current densities of 0.1 C and 0.5 C.

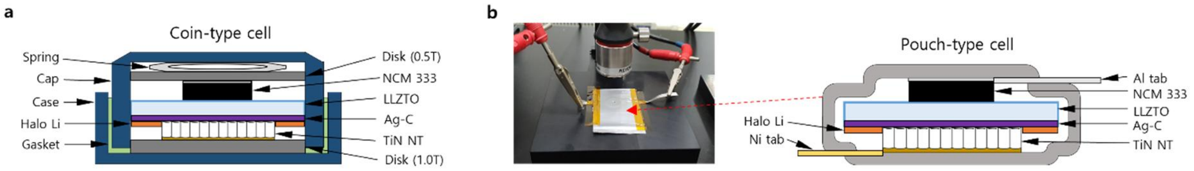

Supplementary Fig. 15. a, Schematic of the coin-cell configuration used for the electrochemical characterizations. **b,** Schematic of the pouch-cell configuration used for the thickness change analysis during the charge/discharge process.

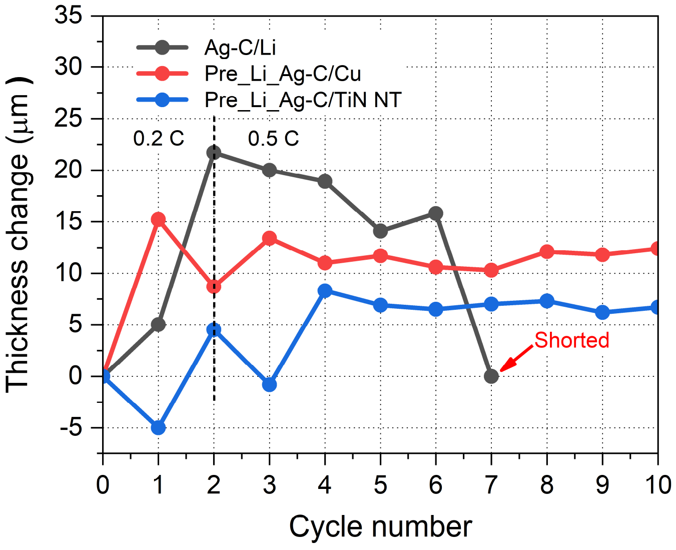

Supplementary Fig. 16. The thickness change of full-cells with different interlayer and anode during lithiation/delithiation as a function of cycle number.

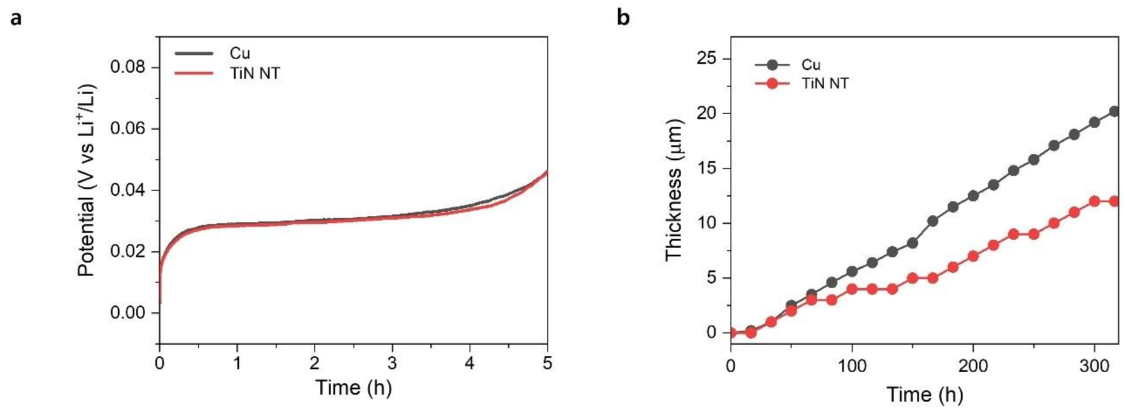

Supplementary Fig. 17. a, Chare voltage profile of Cu and TiN NT incorporated symmetric cells. **b,** Corresponding thickness changes of two symmetric cells as a function of charging time.

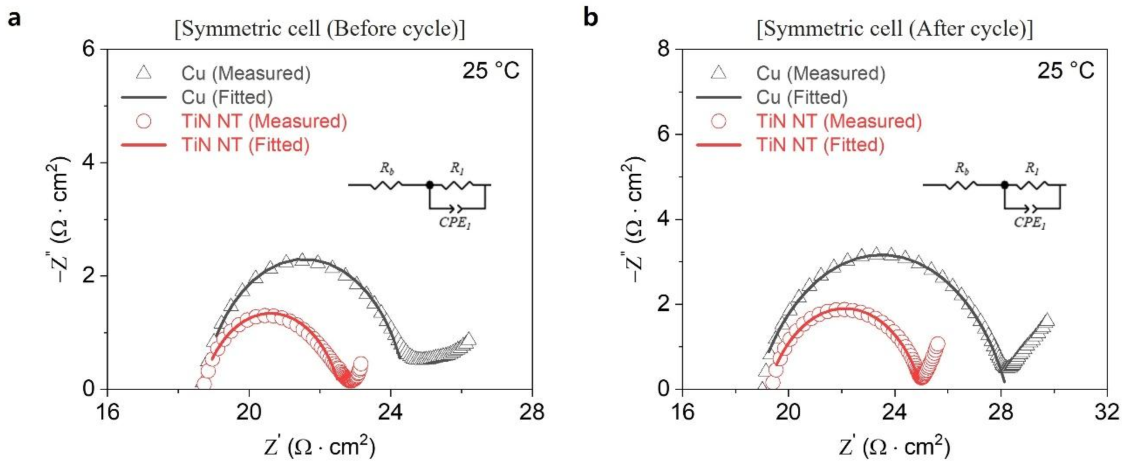

Supplementary Fig. 18. Nyquist plots of AC-impedance spectra obtained from the symmetric cells with different anodes (Cu vs. TiN NT) before and after cycle (inset: equivalent circuit). **a,** Before cycle and **b,** After cycle.

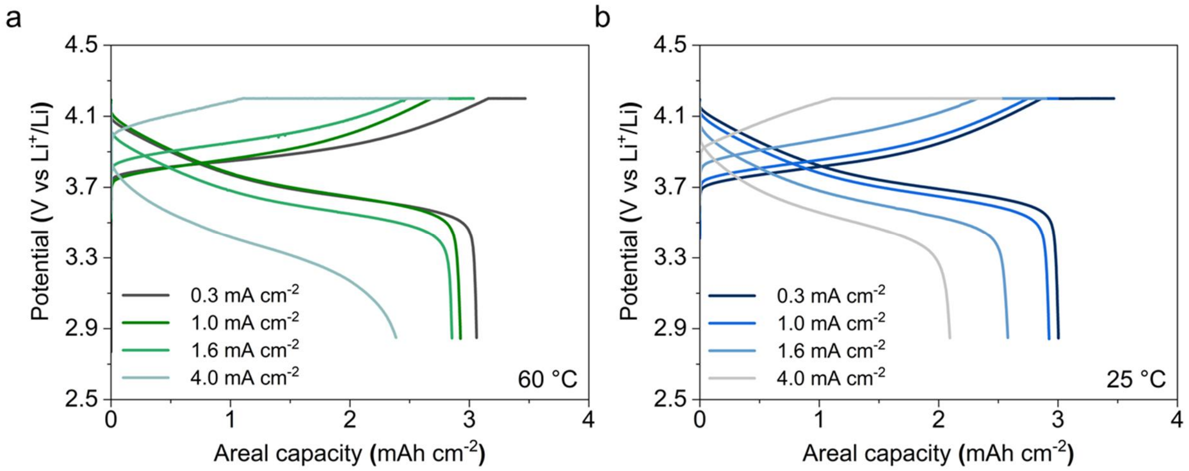

Supplementary Fig. 19. a-b, Charge/discharge voltage profiles of the TiN NT|Pre_Li_Ag-C/LLZTO|IL/NCM333 full-cell with current density ranging from 0.3 to 4.0 mA cm^{-2} at a) 60 ℃, and b) 25 ℃.

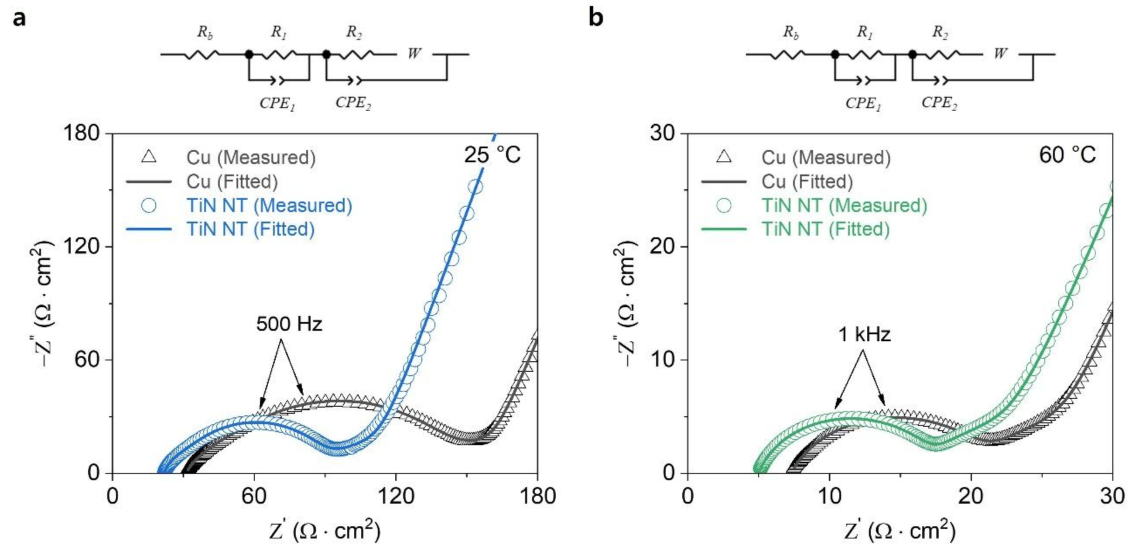

Supplementary Fig. 20. Nyquist plots of AC-impedance spectra obtained from the AFSSB full-cells with different anodes (Cu vs. TiN NT) at different temperature conditions (inset: equivalent circuit). **a,** 25 ℃ and **b,** 60 ℃.

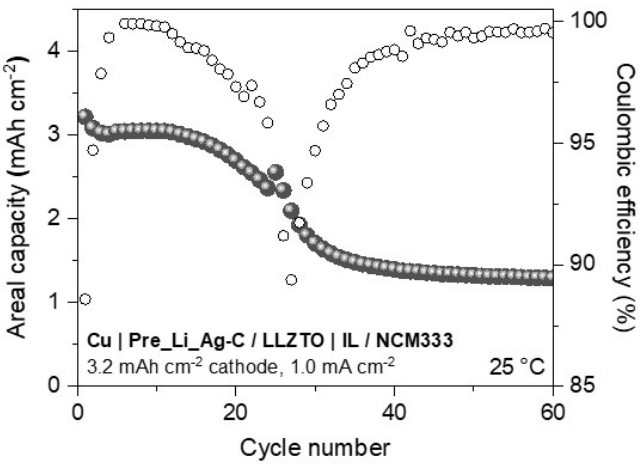

Supplementary Fig. 21. Cycling performance of the Cu|Pre_Li_Ag-C/LLZTO|IL/NCM333 full-cell at 25 °C.

Supplementary references

S1. Tsai, C. -L. et al. A garnet structure-based all-solid-state Li battery without interface modification: resolving incompatibility issues on positive electrodes. *Sustainable Energy Fuels* **3**, 280 (2019).

S2. Ruan, Y. et al. Acid induced conversion towards a robust and lithiophilic interface for Li-$Li_7La_3Zr_2O_{12}$ solid-state batteries. *J. Mater. Chem. A* **7**, 14565 (2019).

S3. Luo, W. et al. Reducing Interfacial Resistance between Garnet-Structured Solid-State Electrolyte and Li-Metal Anode by a Germanium Layer. *Adv. Mater.* **29**, 1606042 (2017).

S4. Chen, S. et al. All-Solid-State Batteries with a Limited Lithium Metal Anode at Room Temperature using a Garnet-Based Electrolyte. *Adv. Mater.* **33**, 2002325 (2021).

S5. Yan, Y. et al. In Situ Polyemerization Permeated Three-Dimensional Li^+-Percolated Porous Oxide Ceramic Framework Boosting All Solid-State Lithium Metal Battery. *Adv. Sci.* **8**, 2003887 (2021).

S6. Huo, H. et al. In-situ formed Li_2CO_3-free garnet/Li interface by rapid acid treatment for dendrite-free solid-state batteries. *Nano Energy* **61**, 119-125 (2019).

S7. Lou, J. et al. Achieving efficient and stable interface between metallic lithium and garnet-type solid electrolyte through a thin indium tin oxide interlayer. *J. Power Sources* **448**, 227440 (2020).

S8. Huo, H. et al. Design of a mixed conductive garnet/Li interface for dendrite-free soild lithium metal batteries. *Energy Environ. Sci.* **13**, 127 (2020).

S9. Shao, Y. et al. Drawing a Soft Interface: An Effective Interfacial Modification Strategy

for Garnet-Type Solid-State Li Batteries. *ACS Energy Lett.* **3**, 1212-1218 (2018).

S10. Lu, G. et al. Built-in superionic conductive phases enabling dendrite-free, long lifespan and high specific capacity composite lithium for stable solid-state lithium batteries. *Energy Environ. Sci.* **16**, 1049 (2023).

S11. Ohta, S., Kobayashi, T., Seki, J. & Asaoka, T. Electrochemical performance of an all-solid-state lithium ion battery with garnet-type oxide electrolyte, *J. Power Sources* **202**, 332-335 (2012).

S12. Yan, C. et al. Garnet-rich composite solid electrolytes for dendrite-free, high-rate, solid-state lithium-metal batteries. *Energy Storage Mater.* **26**, 448-456 (2020).

S13. Chen, H. et al. Improved Interface Stability and Room-Temperature Performance of Solid-State Lithium Batteries by Integrating Cathode/Electrolyte and Graphite Coating. *ACS Appl. Mater. Interfaces* **12**, 15120-15127 (2020).

S14. Xu, H. et al. Li_3N-Modified Garnet Electrolyte for All-Solid-State Lithium Metal Batteries Operated at 40 °C. *Nano Lett.* **18**, 7414-7418 (2018).

S15. Han, F. et al. Interphase Engineering Enabled All-Ceramic Lithium Battery. *Joule* **2**, 497-508 (2018).

S16. Su, J. et al. Overcoming the abnormal grain growth in Ga-doped $Li_7La_3Zr_2O_{12}$ to enhance the electrochemical stability against Li metal. *Ceram. Int.* **45**, 14991-14996 (2019).

S17. Yamamoto, T. et al. A Li-free inverted-stack all-solid-state thin film battery using crystalline cathode material. *Electrochem. Commun.* **105**, 106494 (2019).

S18. Wang, M. J., Carmona, E., Gupta, A., Albertus, P. & Sakamoto, J. Enabling "lithium-

free" manufacturing of pure lithium metal solid-state batteries through in situ plating. *Nat. Commun.* **11**, 5201 (2020).

S19. Zegeye, T. A. et al. Ultrathin $Li_{6.75}La_3Zr_{1.75}Ta_{0.25}O_{12}$-Based Composite Solid Electrolytes Laminated on Anode and Cathode Surfaces for Anode-free Lithium Metal Batteries. *ACS Appl. Energy Mater.* **3**, 11713-11723 (2020).

S20. Kim, J. -S. et al. Surface engineering of inorganic solid-state electrolytes via interlayers strategy for developing long-cycling quasi-all-solid-state lithium batteries. *Nat. Commun.* **14**, 782 (2023).
