# Supplementary material for: Near-strain-free anode architecture enabled by interfacial diffusion creep for initial-anode-free quasi-solid-state batteries
Source: Nat Commun. 2024 Apr 27;15:3586. doi: 10.1038/s41467-024-48021-w (PMC11055892; doi:10.1038/s41467-024-48021-w)
Supplement: Supplementary file 3 — Description of Additional Supplementary Files [file 41467_2024_48021_MOESM3_ESM.pdf]

Description of Additional Supplementary Files

File Name: Supplementary Video 1

Description: In situ OM analysis of the Li propagation.

In situ OM video of the Ag-C layer during the charging/discharging process.
